# Comorbid depression among adults with heart failure in Ethiopia: a hospital-based cross-sectional study

**DOI:** 10.1186/s12888-024-05748-6

**Published:** 2024-04-25

**Authors:** Henok Mulugeta, Peter M. Sinclair, Amanda Wilson

**Affiliations:** 1 Department of Nursing, College of Health Sciences, Debre Markos University, Debre Markos, Amhara Region Ethiopia; 2 School of Nursing and Midwifery, Faculty of Health, University of Technology Sydney, Sydney, NSW Australia

**Keywords:** Depression, Heart failure, PHQ-9, Ethiopia

## Abstract

**Background:**

Depression is a common comorbidity in adults with heart failure. It is associated with poor clinical outcomes, including decreased health-related quality of life and increased morbidity and mortality. There is a lack of data concerning the extent of this issue in Ethiopia. Consequently, this study aimed to assess the prevalence of comorbid depression and associated factors among adults living with heart failure in Ethiopia.

**Methods:**

A hospital-based cross-sectional study was conducted at the cardiac outpatient clinics of two selected specialist public hospitals in Addis Ababa, Ethiopia: St. Paul’s Hospital Millennium Medical College and St. Peter Specialized Hospital. An interviewer-administered questionnaire was used to collect data from 383 adults with heart failure who attended the clinics and met the inclusion criteria. Depression was measured using the Patient Health Questionnaire (PHQ-9). A binary logistic regression model was fitted to identify factors associated with depression. All statistical analyses were conducted using STATA version 17 software.

**Results:**

The mean age of the participants was 55 years. On average, participants had moderate depression, as indicated by the mean PHQ-9 score of 11.02 ± 6.14, and 217 (56.6%, 95%CI 51.53–61.68) had comorbid depression. Significant associations with depression were observed among participants who were female (AOR: 2.31, 95%CI:1.30–4.08), had comorbid diabetes mellitus (AOR: 3.16, 95%CI: 1.47–6.82), were classified as New York Heart Association (NYHA) class IV (AOR: 3.59, 95%CI: 1.05–12.30), reported poor levels of social support (AOR: 6.04, 95%CI: 2.97–12.32), and took more than five medications per day (AOR: 5.26, 95%CI: 2.72–10.18).

**Conclusions:**

This study indicates that over half of all adults with heart failure in Ethiopia have comorbid depression, influenced by several factors. The findings have significant implications in terms of treatment outcomes and quality of life. More research in the area, including interventional and qualitative studies, and consideration of multifaceted approaches, such as psychosocial interventions, are needed to reduce the burden of comorbid depression in this population.

## Introduction

Heart failure (HF) is a global major public health problem with high prevalence and mortality rates [1, 2]. It affects more than 64 million people worldwide [3], and this figure is expected to rise over the next few decades due to ageing and population growth [4, 5]. Globally, the annual cost of HF is estimated to be US$108 billion, and this cost will continue to grow as the prevalence of HF increases [6]. Hospital-based studies show that HF is a serious health concern and its prevalence is increasing in sub-Saharan countries, including Ethiopia [7, 8].

Living with HF is challenging due to the progressive and unpredictable nature of the disease. Adults with HF are more prone to develop negative emotional conditions compared to the general population [9, 10]. Comorbid depression is a prevalent mental health condition in this population and is associated with negative outcomes. It is characterised by persistent feelings of unhappiness, low self-worth and lack of interest in daily activity with symptoms for at least two weeks based on DSM-5 diagnostic criteria [11]. A recent meta-analysis of 149 studies found that 41.9% of adults with HF have any severity of depression, and 28.1% have moderate-to-severe depression [12]. Similarly, the pooled prevalence depressive symptoms in adults with HF in China, based on a meta-analysis of 53 studies, was 43% [13].

Comorbid depression in HF is five times more common than the general population and is an independent predictor of repeated hospital admissions and mortality [14]. Depression can worsen physical symptoms and impair self-care and treatment adherence [15]. Many factors contribute to comorbid depression in adults with HF including age, gender, severity of symptoms, socio-economic status, social support, and hospitalisation [16–18]. Depression is also correlated with poor health-related quality of life [19–21]. It worsens HF symptoms, impairs treatment adherence, reduces self-care practice, and has a double socio-economic impact [22]. Despite the high prevalence of depression and its negative impact on adults with HF, it is often unrecognised and under-treated by health professionals [23, 24].

While several studies have demonstrated the prevalence of comorbid depression among adults with HF in low- and middle-income countries, there is a notable lack of data regarding the extent of this issue in Ethiopia. The current study not only aimed to provide an updated prevalence estimate using rigorous methodologies, but also examined previously unidentified factors associated with depression among adults with HF attending follow-up appointments at two tertiary-level government hospitals in Addis Ababa, the capital of Ethiopia. The findings will provide evidence for health policymakers to identify effective interventions to improve the mental health of adults with HF, ultimately enhancing the prognosis of HF in this population.

## Methods

### Study setting and period

The survey was conducted between 21 Nov 2022 to 22 Jan 2023 at two cardiac outpatient clinics of two government hospitals in Addis Ababa, the capital of Ethiopia: St. Paul’s Hospital Millennium Medical College and St. Peter Specialized Hospital. St Paul’s Hospital Millennium Medical College was established by a decree of the Council of Ministers in 2010, and the hospital was originally founded by the late Emperor Haile Selassie in 1968. The college has over 2800 clinical, academic, and administrative and support staffs who are involved in providing comprehensive tertiary-level care. The hospital boasts over 700 inpatient beds and serves an average of 1200 emergency and outpatient clients daily. St. Peter Specialized Hospital is one of the largest public hospitals in Addis Ababa. The hospital was established in 1963 as the first TB consortium in the country. It offers tertiary-level medical services and education, with around 350 inpatient beds and a catheterization laboratory (Cath lab) for cardiovascular care and interventions, along with 4 ICU beds. These hospitals are the largest tertiary-level teaching hospitals in Ethiopia and receive referred cases from many hospitals across the country. Currently, they collaborate to provide care for people with cardiac conditions, and each hospital typically sees an average of 30 adults with HF per week in their outpatient clinics.

### Study design

A hospital-based cross-sectional study design was conducted.

### Study population

Adults with HF attending a follow-up appointment at the outpatient cardiac clinic in either of the two hospitals during the data collection period.

### Eligibility criteria

The inclusion criteria included individuals over the age of 18 years with a confirmed diagnosis of HF (clinically using Framingham criteria or Echocardiography), who attended follow-up appointments at the outpatient cardiac clinic for at least three months to ensure that they had established ongoing care for their condition, enabling a more holistic assessment of factors affecting depression. Anyone unable or unwilling to provide informed consent was excluded. Additionally, anyone taking antidepressants were also excluded from participating.

### Sample size and sampling procedure

The sample size was determined using a single proportion formula for a finite population, given by N= (Zα/2)^2^*P (1-P)^2^/D^2^) [25], with an assumption of a 95% confidence interval, a marginal error (d) of 5% and a 51% prevalence (P) of depression among adults with HF in Northwest Ethiopia [26]. Therefore, N= {(1.96)^2^*0.511(1-0.511)}/0.05^2^=383 adults with HF. A consecutive sampling technique was used to recruit eligible adults with HF attending the outpatient cardiac clinics until the required sample size was achieved.

## Variables

### Dependent variable

- Depression (Yes/No).

### Independent variables

- Socio-demographic characteristics included: age, sex, educational status, marital status, residence, employment status, social support, health insurance.
- Clinical and other related characteristics included: comorbidity, New York Heart Association (NYHA) class, duration of illness, history of hospitalisation, number of medications taken each day, family history of heart failure, overall health perception.

### Operational definitions and definitions of terms

#### Adult

over 18 years of age.

#### Heart failure

the inability of the heart to effectively pump blood as evidenced by either signs and symptoms based on Framingham criteria or reduced ejection fraction (< 40%) [27, 28].

#### General Health Perception

a representation of all health concepts that determine general satisfaction with life using one global question that asks respondents to rate their overall health on a Likert scale as “excellent”, “very good”, “fair”, or “poor” [29].

#### Community health insurance (CHI)

a health insurance program in Ethiopia designed to provide affordable healthcare coverage to community members.

**The severity of heart failure**: The New York Heart Association criteria (NYHA) was used to classify the severity of heart failure as Class I: no limitation during ordinary activity, Class II: slight limitation during ordinary activity, Class III: marked limitation of normal activities without symptoms at rest, or Class IV: unable to undertake physical activity without symptoms and symptoms may be present at rest [30].

#### Depression

An individual was considered to have depression if their Patient Health Questionnaire (PHQ-9) score was ≥ 10, with higher scores indicating more severe depression [18, 31, 32].

### Data collection procedure and instruments

Trained research assistants (RA), who were cardiovascular nurses working at the facilities, contacted eligible adults with HF attending routine follow-up visits at the outpatient cardiac clinic of each hospital. They explained the purpose of the study and obtained informed consent from those who agreed to participate. The RA collected data from each participant using an interviewer-administered questionnaire in a quiet room within the hospital. The questionnaire consisted of three parts: The first part focused on sociodemographic and clinical characteristics; the second part addressed social support; and the third part assessed depression. The sociodemographic and clinical characteristics were assessed using a 19-item questionnaire. This questionnaire asked about age, sex, marital status, employment status, residence, educational level, health insurance, family history of HF, hospitalisation history, comorbidities, duration of illness, NYHA class, number of medications and general health perception. Data regarding comorbidities and the severity of HF (NYHA class) were extracted from the patient’s medical charts. The Oslo Social Support Scale (OSSS-3) was used to assess the level of social support. It consists of three items all focusing on accessibility of practical help: the number of close confidants; the sense of concern from other people; and relationships with neighbours. The total score ranges from 3 to 14, with high values representing strong support and low values representing poor social support [33]. It has good construct and predictive validity and a good internal consistency having a Cronbach alpha of 0.91 [34]. Depression was measured using the Patient Health Questionnaire (PHQ-9), which is a 9-item tool based on DSM-IV criteria where item scores range from 0 (not at all) to 3 (nearly every day). The total score ranges from 0 to 27 to measure depression severity, with higher scores indicating a higher likelihood of depression. Each item requires participants to rate the frequency of a depressive symptom experienced in two weeks prior to the assessment. The PHQ-9 is a reliable and valid instrument for detecting major depressive disorder among Ethiopian adults with chronic conditions in outpatient settings [35, 36]. Data collection was carried out by trained health professionals working in the outpatient cardiac clinics of each hospital, with two supervisors overseeing the data collection process. The collected data were reviewed daily to ensure completeness and clarity.

### Data analysis

Data were cleaned and entered into Epi-Data version 3.1, and then exported to STATA Version 17 for analysis [37]. Descriptive analyses, including frequency, mean and standard deviation, were performed to describe the socio-demographic, clinical and other characteristics of the participants. A binary logistic regression model was fitted to identify factors associated with depression. A simple bivariable analysis was performed to test the association between each independent variable and the dependent variable. All independent variables with a p-value of less than 0.25 in the bivariable analysis were considered eligible for further analysis in the multivariable logistic regression model. A statistically significant association was declared when p-value was 0.05 or below. Multicollinearity was assessed by calculating the variance inflation factor (VIF), while the adequacy of model fitness was checked using the Hosmer-Lemeshow goodness-of-fit test. The results were presented in text, tables and graphs based on the data types.

## Results

### Sociodemographic characteristics of the study participants

A total of 383 adults with HF participated in this study, with a response rate of 100%. Of these, 184 (48.04%) were male, with a mean age of 55.1 ± 15.38 years. A total of 196 (51.17%) participants were married, and 184 (48.04%) were employed. Nearly three-fourths, or, 286 (74.67%), resided in urban areas, and 114 (29.77%) had low levels or no formal education. Additionally, nearly three-fourths, or 276 (72.06%) of the study participants were enrolled in the community health insurance (CHI) scheme. Details of the socio-demographic characteristics of the participants are summarised in Table 1.

**Table 1:** Sociodemographic characteristics of people with HF in Ethiopia, 2023 (*n* = 383)

Variables	Category	Frequency (%)
Age	18–39	74 (19.32)
	40–69	222 (57.96)
	≥70	87 (22.72)
Sex	Male	184 (48.04)
	Female	199 (51.96)
Marital status	Single	65 (16.97)
	Married	196 (51.17)
	Divorced	33 (8.62)
	Widowed	69 (18.02)
	Separated	20 (5.22)
Employment status	Employed	184 (48.04)
	Unemployed	199 (51.96)
Residence	Urban	286 (74.67)
	Rural	97 (25.33)
Educational level	Low or no education	114 (29.77)
	Primary education	108 (28.20)
	Secondary education	87 (22.72)
	College and above	74 (19.32)
Community health insurance	Yes	276 (72.06)
	No	107 (27.94)

### Clinical characteristics of the study participants

Most participants (87.73) reported no family history of HF, while 132 (34.46%) had a history of hospitalisation in the previous twelve months. The mean duration of HF among participants was 2.5 years, and the majority, 276 (72.06%) took less than five medications daily. Of the participants, 169 (44.13%) participants had comorbid hypertension and 80 (20.89%) had comorbid diabetes. Additionally, 135 (35.25%) participants were classified in NYHA class I, and 100 (26.11%) had poor General Health Perception. The mean social support score of the participants on OSLO-3 was 8.98 ± 2.94. Details of the clinical characteristics are summarised in Table 2.

**Table 2:** Clinical characteristics of people with HF in Ethiopia, 2023 (*n* = 383)

Variables	Category	Frequency (%)
Family history of heart failure	No	336 (87.73)
	Yes	47 (12.27)
History of hospitalisation in the last 12 months	No	251 (65.54)
	Yes	132 (34.46)
Comorbidities		
Hypertension	Yes	169 (44.13)
Diabetes	Yes	80 (20.89)
Kidney disease	Yes	29 (7.57)
COPD and asthma	Yes	11 (2.87)
Cancer	Yes	3 (0.78)
HIV/AIDS	Yes	19 (4.96)
Length of time since HF diagnosis	< 1year	65 (16.97)
	1–5 years	154 (40.21)
	5–10 years	107 (27.94)
	10–15 years	34 (8.88)
	> 15 years	23 (6.01)
Medications taken daily	< 5	276 (72.06)
	≥ 5	107 (27.94)
NYHA class	Class I	135 (35.25)
	Class II	117 (30.55)
	Class III	93 (24.28)
	Class IV	38 (9.92)
General health perception	Excellent	32 (8.36)
	Very good	102 (26.63)
	Fair	149 (38.90)
	Poor	100 (26.11)
Social support scores	Mean ± SD	8.98 ± 2.94

### The PHQ-9 scale scores and the prevalence of depression among participants

Participants were asked about their experiences in the two weeks before the interview: 136 (35.51%) reported little interest or pleasure in doing things for several days, while 117 (30.55%) had trouble falling or staying asleep or sleeping too much over several days. Additionally, 103 (26.89) had poor appetite, and 99 (25.85) reported trouble falling or staying asleep or sleeping too much more than half the days. Only a small percentage (5.48%) thought they would be better off dead or considered hurting themselves. Descriptive statistics for individual items on the PHQ-9 scale scores are summarised in Table 3.

**Table 3:** Descriptive statistics for individual items of PHQ-9 scale

PHQ-9 items	Frequency (%)			
How often have you bothered by any of the following problems over the last 2 weeks?	Not at all	Several days	More than half the days	Nearly everyday
Little interest or pleasure in doing things	94 (24.54)	136 (35.51)	109 (28.46)	44 (11.49)
Feeling down, depressed, or hopeless	122 (31.85)	125 (32.64)	72 (18.80)	64 (16.71)
Trouble falling or staying asleep, or sleeping too much	109 (28.46)	117 (30.55)	99 (25.85)	58 (15.14)
Feeling tired or having little energy	16 (4.18)	104 (27.15)	123 (32.11)	140 (36.55)
Poor appetite or overeating	107(27.94)	115 (30.03)	103 (26.89)	58 (15.14)
Feeling bad about yourself or that you are a failure or have let yourself or your family down	130 (33.94)	111 (28.98)	66 (17.23)	76 (19.84)
Trouble concentrating on things, such as reading the newspaper or watching television	175 (45.69)	120 (31.33)	62 (16.19)	26 (6.79)
Moving or speaking so slowly that other people could have noticed.	117 (30.55)	120 (31.33)	78 (20.37)	68 (17.75
Thoughts that you would be better off dead, or of hurting yourself	232 (60.57)	77 (20.10)	53 (13.84)	21 (5.48)

**Fig. 1:**
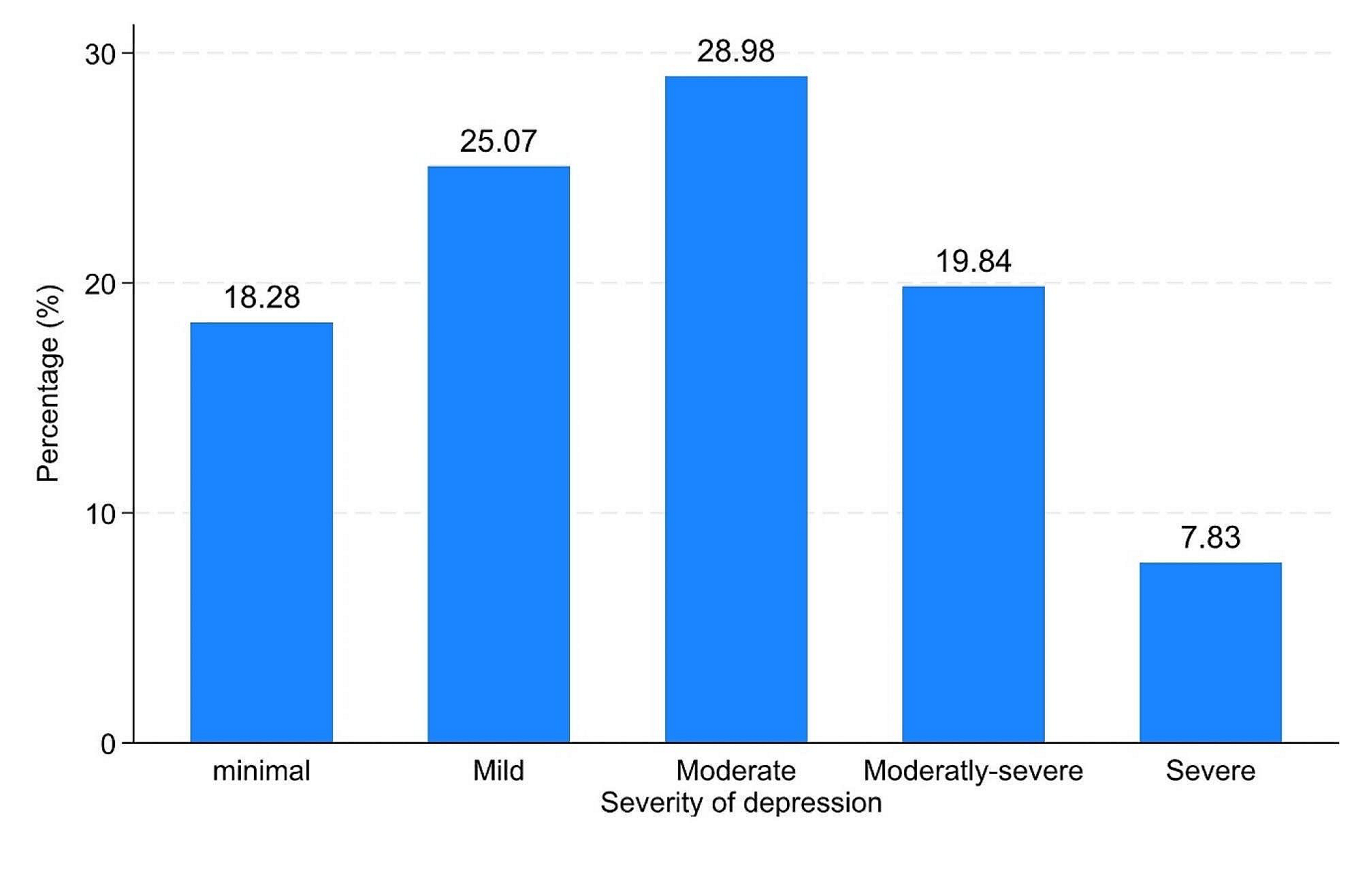
Severity of depression among adults with HF in Ethiopia

Participants’ PHQ-9 scores ranged from 1 to 27 and were categorised as follows: minimal depression (0–4), mild depression [5–9], moderate depression [10–14], moderately severe depression [15–19], and severe depression (20 or higher). The results indicated that 18.28% of participants had minimal depression, 25.07% had mild depression, 28.98% had moderate depression, 19.84% had moderately severe depression, and 7.83% had severe depression (Fig. 1). The mean PHQ-9 score of the participants was 11.02 ± 6.14, signifying that, on average, they had moderate depression. In total, 217 (56.66%) of the participants in this study had comorbid depression.

### Factors associated with comorbid depression in adults with heart failure

The binary logistic regression analysis revealed that several factors, including age, gender, marital status, employment status, educational level, hospitalisation history, diabetes mellitus (DM), chronic obstructive pulmonary disease (COPD), number of medications, NYMA class, social support, and general health perception were significant, with a *p*-value of less than 0.20 during the bivariable analysis. Consequently, these variables were included in the final model. However, in the multivariable logistic regression analysis, only six independent variables (gender, comorbid diabetes, NYHA class, social support, number of medications, and general health perception) showed a significant association with depression (*p* ≤ 0.05).

The odds of comorbid depression were 2.31 (95%CI 1.30,4.08) times higher among females compared to males. Participants with HF and diabetes were 3.16 (95%CI 1.47,6.82) times more likely to be depressed compared to those without diabetes. Additionally, participants taking more than five medications daily had 5.26 (95%CI 2.72,10.18) times higher odds of comorbid depression compared to those taking fewer medications. The binary logistic regression analysis of factors associated with comorbid depression in adults with HF is summarised in Table 4.

**Table 4:** Bivariable and multivariable logistic regression analyses of factors associated with depression in adults with HF in Ethiopia, 2023, (*n* = 383)

Variables	Depression		COR (95%CI)	AOR (95%CI)	P-value
	Yes N (%)	No N (%)			
Age					
18–39	38(17.51)	36(21.69)	1	1	
40–69	124(57.14)	98(59.04)	1.20(0.71,2.03)	0.71 (0.34,1.46)	0.346
≥70	55(25.35)	32(19.28)	1.63(0.87,3.06)	0.49 (0.19,1.28)	0.145
Sex					
Male	90(41.47)	94(56.63)	1	1	
Female	127(58.53)	72(43.37)	1.84(1.22,2.77)	2.31 (1.30,4.08)	0.004*
Marital status					
Single	34(15.67)	31(18.67)	0.88(0.50,1.54)	1.09(0.53,2.25)	0.808
Married	109(50.23)	87(52.41)	1	1	
Divorced	23(10.60)	10(6.02)	1.84(0.83,4.06)	1.53(0.54,4.29)	0.423
Widowed	41(18.89)	28(16.87)	1.17(0.67,2.04)	0.81(0.37,1.78)	0.603
Separated	10(4.61)	10(6.02)	0.80(0.32,2.00)	0.51(0.16,1.65)	0.260
Educational level					
Illiterate	71(32.72)	43(25.90)	1	1	
Primary	69(31.80)	39(23.49)	1.07(0.62,1.85)	0.76(0.37,1.60)	0.458
Secondary	40(18.43)	47(28.31)	0.52(0.29,0.91)	0.62(0.29,1.30)	0.204
College & above	37(17.05)	37(22.29)	0.61(0.33,1.10)	0.96(0.41,2.24)	0.928
Diabetes mellitus					
No	150(69.12)	153(92.17)	1	1	
Yes	67(30.88)	13(7.83)	5.26(2.79,9.92)	3.16(1.47,6.82)	0.003*
COPD					
No	208(95.85)	164(98.80)	1	1	
Yes	9(4.15)	2(1.20)	3.55(0.76,16.65)	2.43(0.41,14.49)	0.329
NYHA class					
Class I	62(28.57)	73(43.98)	1	1	
Class II	62(28.11)	56(33.73)	1.28(0.78,2.11)	1.00(0.54,1.87)	0.997
Class III	62(28.11)	32(19.28)	2.24(1.30,3.78)	1.59(0.78,3.23)	0.204
Class IV	33(15.21)	5(3.01)	7.77(2.86,21.12)	3.59(1.05,12.30)	0.042*
Social support					
Poor	123(56.68)	35(21.08)	7.14(4.05,12.57)	6.04(2.97,12.32)	0.001*
Moderate	62(28.57)	66(39.76)	1.91(1.10,3.29)	1.78(0.89,3.57)	0.103
Strong	32(14.75)	65(39.16)	1	1	
Employment status					
Employed	87(40.09)	97(58.43)	1	1	
Unemployed	130(59.91)	69(41.57)	2.10(1.39,3.17)	1.18(0.67,2.09)	0.562
Hospitalisation History					
No	134(61.75)	117(70.48)	1	1	
Yes	83(38.25)	49(29.52)	1.48(0.96,2.28)	1.05(0.60,1.83)	0.863
Number of medications daily					
<5	133(61.29)	143(86.14)	1	1	
≥5	84(38.71)	23(13.86)	3.93(2.34,6.59)	5.26(2.72,10.18)	0.001*
Health perception					
Excellent	11(5.07)	21(12.65)	1	1	
Very good	36(16.59)	66(39.76)	1.04(0.45,2.40)	1.24(0.44,3.44)	0.684
Fair	93(42.86)	56(33.73)	3.17(1.42,7.07)	3.50(1.29,9.52)	0.014*
Poor	77(35.48)	23(13.86)	6.39(2.69,15.19)	4.74(1.64,13.73)	0.004*

*Note*: - N = Frequency; COPD: Chronic obstructive pulmonary disease; CI: Confidence Interval; COR: Cruds Odds Ratio; AOR: Adjusted Odds Ratio; NYHA: New York Heart Association

## Discussion

Comorbid depression in adults with HF is a relatively common problem which is associated with increased mortality and morbidity [38, 39]. Given this context, this study aimed to determine the prevalence of comorbid depression and identify its associated factors among adults with HF in Ethiopia.

This study revealed that the prevalence of comorbid depression was 56.66% (95%CI 51.53, 61.68) among adults with HF, which aligns with a similar study conducted Northwest Ethiopia [26]. Our previous systematic review and meta-analysis also demonstrated a comparable pooled prevalence of depression among adults with HF in low-and middle-income countries (LMICs) [40]. These findings demonstrate that depression in adults with HF is under diagnosed and under treated in LMICs, including Ethiopia, indicating a higher prevalence of depression compared to developed countries. For instance, the prevalence of depression in adults with HF was 17.3% in the USA [17], 28.6% in the UK [41], 29.7% in Spain [42], 41% in the Netherlands [43]. The higher prevalence rate in our study could be due limited access to healthcare, including mental health services [44, 45], which might hinder the early identification and treatment of depression. Developed countries generally have more stable economies, which contributes to reduced stress and anxiety, thereby lowering the risk of depression [46]. Conversely, the prevalence in this study is lower than in similar studies conducted in Jordan (65%) [47], Pakistan (66%) [48], South Korea (67.9%) [49], and Indonesia (85.2%) [50]. This discrepancy might be due to differences in methodology, measurement tools, the definition of depression, sample size, and the socioeconomic conditions of the study participants. These results support the notions that researchers and the audience should take contextual and methodological factors into consideration while interpreting and comparing prevalence rates across different studies.

In this study, females had a higher prevalence of depression than males. This finding is consistent with the results of other studies [17, 51, 52]. A meta-analysis on the global prevalence of depression in adults with HF also found a higher prevalence of depression among women compared to men [12]. Females are more susceptible to stressors related to caregiving and family responsibilities, which could contribute to higher rates of depression [53]. Hormonal differences between men and women may also play a role in the development and severity of depression [54, 55]. This underscores the need to consider gender-specific interventions aimed at reducing the prevalence of depression among female patients with HF.

Our study showed that adults with diabetes had a significantly higher prevalence of depression compared to those without diabetes. Similar findings have been observed in other studies [48, 56]. The burden of complications, financial stress, poor glycaemic control, and overall poor health status among adults with both HF and diabetes can be overwhelming and stressful. This can lead to feelings of frustration and hopelessness, which, in turn, may contribute to the development of depression in this population [57–59]. Adults with NYHA class IV had significantly higher prevalence of depression compared to those with NYHA class I, which is consistent with previous similar studies [18, 24, 60, 61]. Adults with higher NYHA classes experience more severe symptoms and limitations, which can increase the risk of depression [62]. These finding collectively emphasize the importance of developing effective interventions for addressing depression for people living with both HF and diabetes, especially those with advanced diseases stages.

Consistent with previous studies [16, 26, 63, 64], our findings demonstrated a significant association between social support and depression. People with poor social support were more likely to be depressed compared to those with strong social support. A recent systematic review also found that social support serves as a protective factor against depression in Western countries [65]. Social support provides both emotional and practical assistance, helping people to cope with the challenges of HF. Those who lack social support may feel isolated, lonely, and overwhelmed [63, 65], which can contribute to the development of depression. This underscores the importance of incorporating strong social support networks into HF care plans aimed at reducing the burden of depression and improving overall well-being.

There was a significant association between the number of medications taken daily and depression. Participants taking more than five medications daily had a higher prevalence of depression. This finding is in line with a systematic review and meta-analysis which found polypharmacy was significantly associated with an increased risk of depression in adults with HF [66]. This could be due to increased side effects and adverse reactions, which can lead to negative emotions, including depression [66, 67]. Considering this finding, careful monitoring of the patients’ medication regimens and educating them about the medication management, including the importance of adherence, and potential side effects, are critical for reducing the influence of polypharmacy on mental health outcomes in people with HF.

Determining the prevalence of comorbid depression and associated factors in adults with HF has important clinical implications. It informs healthcare providers regarding the burden of depression on these populations, thereby improving the development of appropriate care strategies. Addressing comorbid depression in adults with HF may lead to improvements in outcomes such as health-related quality of life, morbidity, and mortality. Identification of various sociodemographic and clinical characteristics as a factor affecting depression could be critical in developing interventions aimed to address the specific needs of individuals with HF.

Although this study provides current evidence on comorbid depression in adults with HF in Ethiopia, it has limitations that should be considered when interpreting its results. This study was cross-sectional in nature, which means it cannot establish causality [68, 69] or determine the relationship between depression and sociodemographic and clinical variables. The study used self-reported data to measure depression and other independent variables, which can be subjective to recall bias and social desirability bias [70]. Dichotomizing PHQ-9 scores may misclassify individuals and ignore small variations in the severity of depression symptoms, which could reduce the sensitivity and depth of the data analysis. Lastly, depressive symptoms may change over time, and this study only captured data at a single point in time. Therefore, longitudinal studies are required to investigate the temporal relationship between variables and to track changes in depressive symptoms over time.

## Conclusion

This study found that more than half of all adults with HF in Ethiopia had comorbid depression. Factors such as gender, comorbid diabetes, NYHA class, social support, number of daily medications, and general health perception were significantly associated with comorbid depression in adults with HF. Healthcare providers should be aware of the high prevalence of depression in this population and should incorporate regular depression screening into their routine care practices. Moreover, effective multimodal interventions, such as psychosocial interventions targeting the improvement of mental health, should be carefully developed to reduce the burden of comorbid depression in this population.

## Data Availability

All the data analysed in this study are available from the corresponding author up on a reasonable request.

## Abbreviations

AOR: Adjusted Odds Ratio
CI: Confidence Interval
COPD: Chronic obstructive pulmonary disease
COR: Crude Odds Ratio
DM: Diabetes Mellitus
DSM-5: Diagnostic and Statistical Manual of Mental Disorders
GHP: General Health Perception
HF: Heart Failure
HRQoL: Health-Related Quality of Life
NYHA: New York Heart Association
OSSS-3: Oslo Social Support Scale
PHQ-9: Patient Health Questionnaire
SE: Standard Error
